# Mapping the research landscape of PET/CT in lymphoma: insights from a bibliometric analysis

**DOI:** 10.3389/fonc.2025.1513296

**Published:** 2025-04-08

**Authors:** Die Zhang, Jianding Peng, Yingjie Zhu, Qiang Gong, Qing Wang, Chaodong Xiang, Hanjian Du, Xiaofei Hu

**Affiliations:** ^1^ Department of Neurology, The First Hospital Affiliated of Army Medical University (Southwest Hospital), Chongqing, China; ^2^ Beijing Tongren Hospital, Capital Medical University, Beijing, China; ^3^ Department of Nuclear Medicine, The First Hospital Affiliated of Army Medical University (Southwest Hospital), Chongqing, China; ^4^ Department of Hematology, The First Hospital Affiliated of Army Medical University (Southwest Hospital), Chongqing, China; ^5^ Institute of Medical Information, Chinese Academy of Medical Sciences & Peking Union Medical College, Beijing, China; ^6^ School of Medicine, Chongqing University, Chongqing, China; ^7^ Department of Neurosurgery, Chongqing Research Center for Glioma Precision Medicine, Chongqing General Hospital, Chongqing University, Chongqing, China

**Keywords:** PET/CT, lymphoma, bibliometric analysis, precision medicine, diagnostic imaging

## Abstract

**Objective:**

This study provides a comprehensive bibliometric analysis of research trends in Positron Emission Tomography/Computed Tomography (PET/CT) applications for lymphoma, aiming to identify key contributors, emerging topics, and collaboration patterns within the field.

**Methods:**

Data from the Web of Science Core Collection (2004–2024) were analyzed. Original articles and reviews in English on PET/CT in lymphoma staging, response assessment, or prognosis were included, while case reports, meeting abstracts, and editorials were excluded. Using CiteSpace, VOSviewer, and Bibliometrix R, we evaluated country/institutional contributions, co-citation networks, keyword trends, and employed linear regression for trend forecasting.

**Results:**

A total of 2,962 papers related to PET/CT and lymphoma were published during the study period. The annual publication volume increased significantly, peaking in 2021 with 281 papers, followed by a decline to 260 in 2023, potentially linked to COVID-19-related research disruptions. The United States and China led in publication volume, contributing over 40% of global publications. Leading institutions included UNICANCER and Assistance Publique Hôpitaux de Paris. Influential authors such as Sally F. Barrington and Michel Meignan were identified. The European Journal of Nuclear Medicine and Molecular Imaging and the Journal of Nuclear Medicine were the top journals in this field. Key research themes included staging, response assessment, prognosis, and the role of PET/CT in personalized treatment approaches.

**Conclusion:**

This bibliometric analysis highlights the significant growth and evolving trends in PET/CT research for lymphoma. The findings underscore the critical role of PET/CT in advancing precision medicine, informing future research directions, and optimizing clinical practices in lymphoma management.

## Introduction

Lymphoma, a diverse group of hematological malignancies, poses significant diagnostic and therapeutic challenges due to its heterogeneity and complexity (1, 2). Accurate staging and response assessment are critical for guiding treatment decisions and predicting patient outcomes in lymphoma (3). In this context, positron emission tomography/computed tomography (PET/CT) has emerged as an invaluable imaging modality in the management of lymphoma. By combining functional PET imaging with anatomical CT data, PET/CT provides a comprehensive evaluation of disease extent, metabolic activity, and treatment response, revolutionizing the approach to lymphoma care (4, 5).

The widespread adoption of PET/CT in the clinical management of lymphoma can be attributed to its numerous advantages. It plays a pivotal role in initial staging, enabling precise delineation of nodal and extranodal involvement, which is critical for appropriate treatment planning (6). Moreover, interim PET/CT scans have become instrumental in monitoring treatment response, allowing for early identification of refractory disease and facilitating risk-adapted therapy (7). At the end of treatment, PET/CT is essential for assessing complete metabolic response, guiding post-treatment surveillance strategies (8). The integration of PET/CT has substantially impacted lymphoma management, leading to improved prognostication, tailored treatment regimens, and ultimately, better patient outcomes (9). Emerging evidence also highlights PET/CT’s role in predicting response to novel immunotherapies (e.g., PD-1 inhibitors), where metabolic shifts on interim PET/CT correlate with T-cell activation (10).

The role of PET/CT in lymphoma continues to evolve, driven by ongoing research and technological advancements. Beyond the ubiquitous 18F-FDG, novel PET tracers targeting specific molecular pathways—such as CXCR4 (68Ga-Pentixafor), CD20 (89Zr-rituximab), and fibroblast activation protein (68Ga-FAPI)—are increasingly explored for subtype-specific imaging and therapy personalization. These advancements holds promise for enhancing diagnostic accuracy and personalizing treatment approaches (11). Additionally, the integration of PET/CT with other imaging modalities, such as PET/MRI, offers the potential for superior lesion characterization and multiparametric evaluation (12). Furthermore, the application of advanced image analysis techniques, including radiomics and artificial intelligence, is poised to unlock new insights from PET/CT data, enabling more precise risk stratification and treatment response prediction (13). Ongoing research is crucial to further refine and optimize the use of PET/CT in lymphoma, leveraging these emerging technologies and analytical approaches.

Bibliometrics, a quantitative method for analyzing scientific literature, provides a powerful tool for identifying research trends, influential publications, collaborations, and emerging topics in a specific field. By employing statistical techniques to examine publication data, bibliometric analysis offers a comprehensive overview of the research landscape, revealing patterns, interconnections, and knowledge gaps (14). In the realm of medical research, bibliometric analyses have proven invaluable in guiding future research directions, fostering collaborations, and informing evidence-based decision-making (15).

While bibliometric studies have been conducted in various oncological domains, including lymphoma (16) and PET/CT imaging (17), a comprehensive bibliometric analysis specifically focused on the research trends of PET/CT in lymphoma is currently lacking. Existing studies have explored broader aspects of lymphoma research or PET/CT applications in general but have not delved into the specific intersection of these two critical areas, which has witnessed substantial growth and advancements in recent years. This bibliometric study aims to fill this gap by providing a comprehensive understanding of the research landscape surrounding PET/CT in lymphoma, and will offer valuable insights to researchers, clinicians, and policymakers, guiding future endeavors in optimizing the application of PET/CT for improved lymphoma management and patient outcomes.

## Methods

### Data source and search strategy

Records related to PET/CT and lymphoma were retrieved from the Web of Science Core Collection (WoSCC) database, covering the period from January 1, 2004, to February 29, 2024. The search strategy employed was as follows: (TS = (PET-CT OR PET/CT)) AND TS = ((Lymphoma* OR lymphatic cancer*)). The document types were limited to original research articles and reviews involving human subjects and preclinical studies (e.g., animal models). The language restriction was set to English. No restrictions were applied to radiotracer types, encompassing both FDG and non-FDG agents (e.g., 68Ga-Pentixafor, 18F-FLT). All searches and data extractions were completed on March 12, 2024.

### Data analysis

The exported documents from the WoSCC database were imported into CiteSpace 6.2.R4 and VOSviewer 1.6.19 for analysis. CiteSpace 6.2.R4 was used for visualizing country/region and institution contributions, co-citation analysis of references, keyword contributions, clustering, timeline clustering, and detecting burst keywords. VOSviewer 1.6.19 was used for visualizing author and co-cited author networks, co-citation analysis of journals, and keyword clustering analysis. The Bibliometrix R package was utilized to display source and thematic dynamics and to visualize the global distribution of publications. Excel 2019 was employed to present the annual publication trends and citation counts and to perform linear regression forecasting of future publication numbers.

## Results

### Annual article production

**Figure 1:**
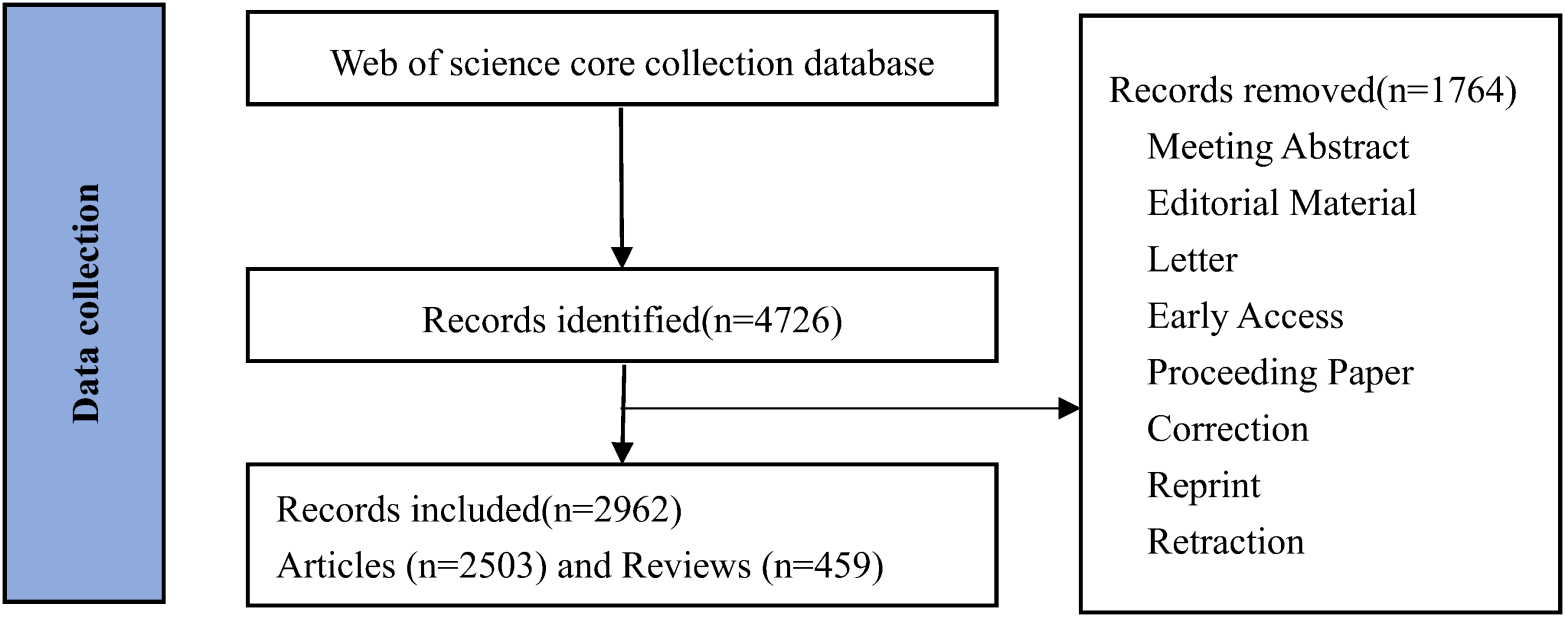
Flow chart of inclusion and exclusion criteria for bibliometrics analysis.

From 2004 to February 29, 2024, a total of 2,962 papers related to PET/CT and lymphoma were published, including 2,503 research articles (84.5%) and 459 review articles (15.5%) (**Figure 1**). According to the statistics: Among all the collected articles, the ratio of prospective studies to retrospective studies is 2:1 (700: 385), and the ratio of clinical studies to preclinical studies is 29:2 (867: 63). A detailed analysis of the literature revealed 70 meta-analyses and 416 systematic reviews. It should be noted that some meta-analyses were categorized as research articles in their respective journals. Among all the research articles, 164 were multicenter studies and 100 were single-center studies. Citation analysis revealed the top 10 cited papers of the two types of studies (**Supplementary Tables 1**, **2**).

**Figure 2:**
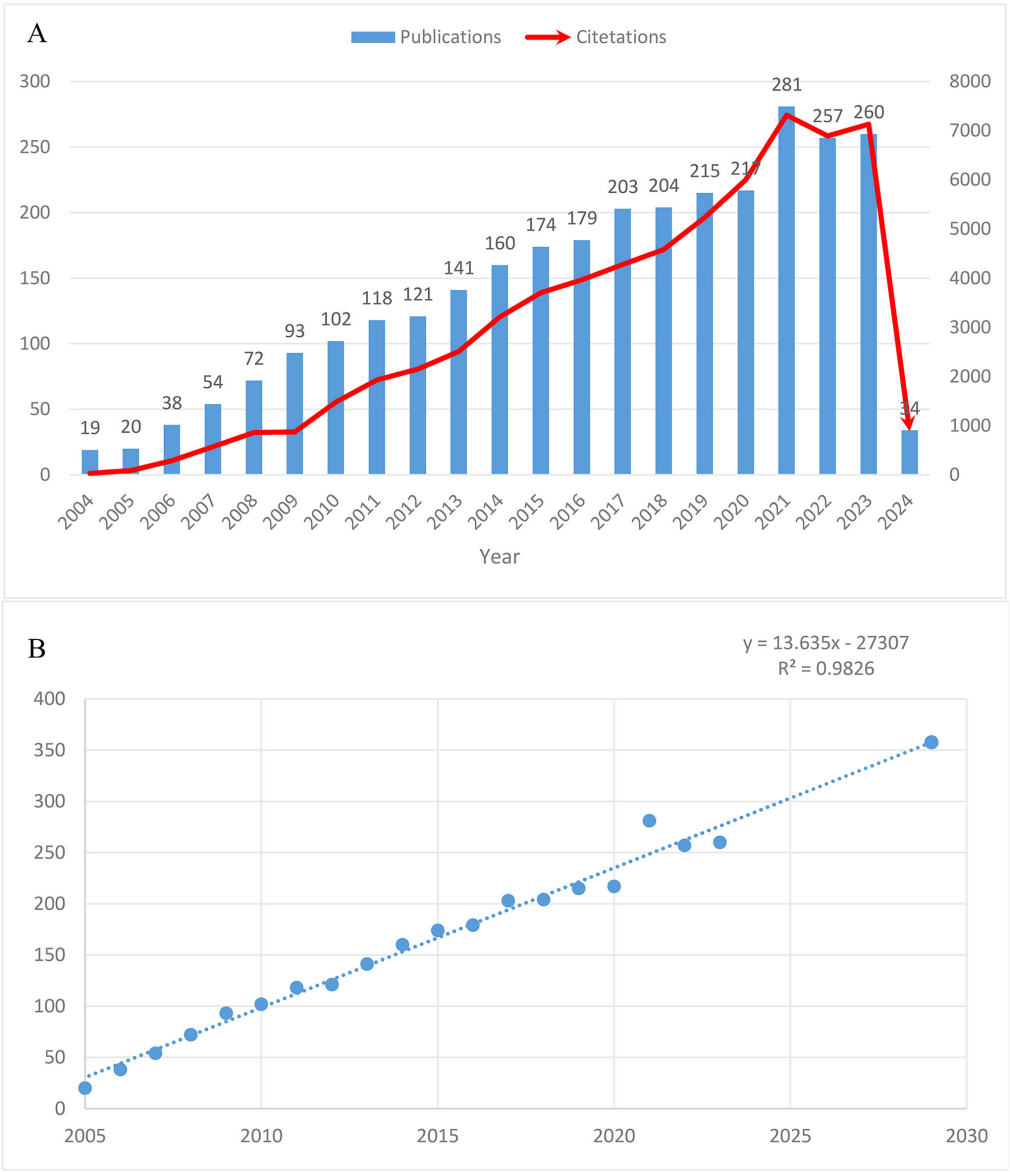
Trends in the growth of publications worldwide. **(A)** Trends in the growth of publications worldwide from 2004 to 2024. **(B)** The estimated annual publication count to 2030.

Notably, the number of published papers showed a dynamic change over the past decade, reflecting the overall development trend in this field. As shown in **Figure 2A**, the annual publication volume steadily increased from 2004 to 2020, with a significant surge to 281 papers in 2021. Subsequently, a slight decline to around 260 papers was observed in 2022 and 2023. Correspondingly, the citation counts increased from 29 in 2004 to 6,004 in 2020, with a sharp rise to 7,318 in 2021. As of February 29, 2024, 34 papers had been published, and the estimated annual publication volume for 2024 is projected to reach 290, with the number expected to exceed 350 by 2030 (**Figure 2B**).

### Country and regional contributions

**Figure 3:**
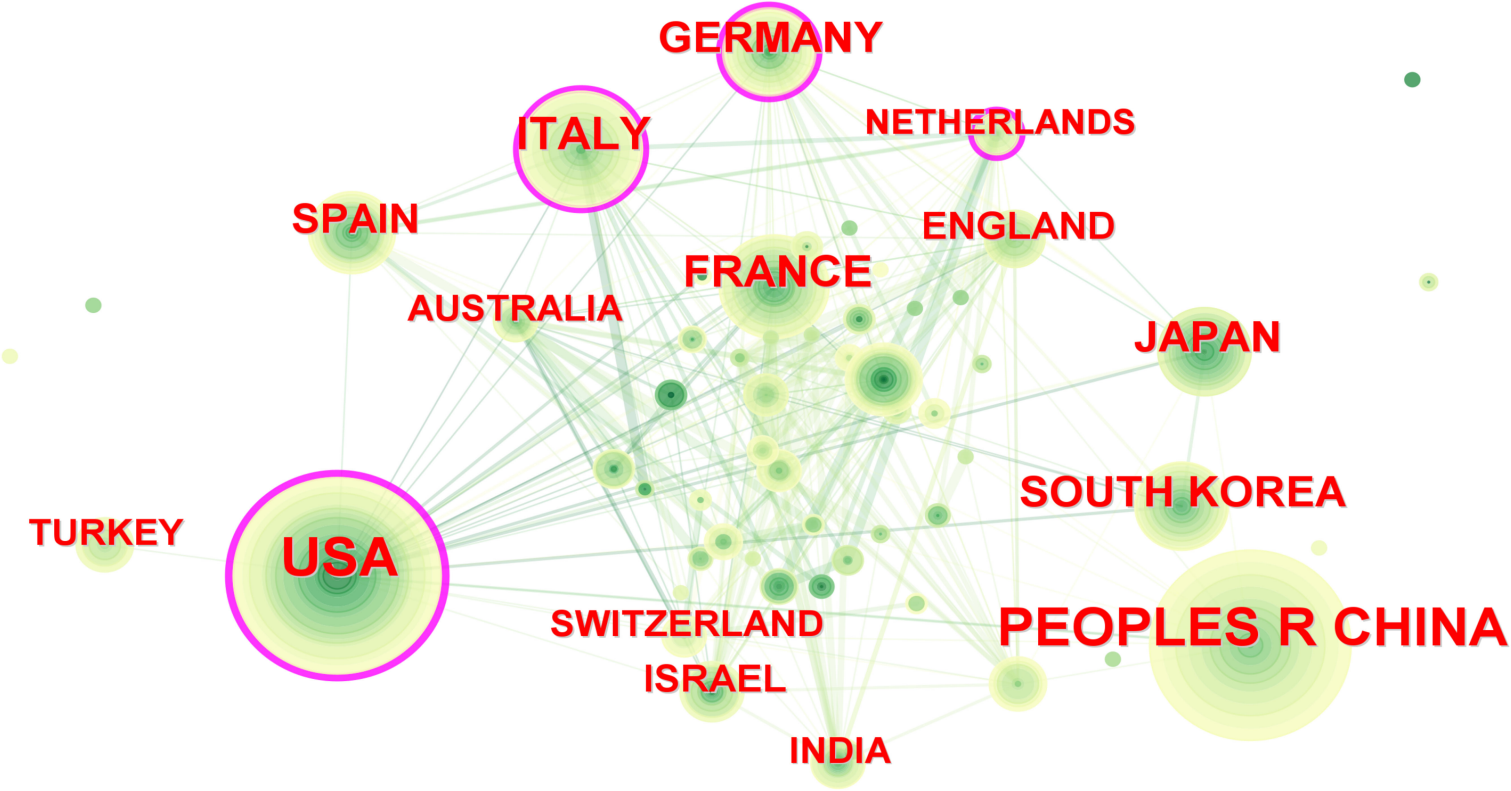
Inter-country cooperation network map. The size of the nodes indicates the number of publications, and the lines indicate collaboration frequency.

In the past decade, 72 countries/regions conducted research on PET/CT in lymphoma. The United States led with 684 publications, followed by China with 577 articles, contributing to over 40% of global publications (**Table 1**). Italy, France, and Germany also made significant contributions, with over 200 articles each, ranking third to fifth, respectively. Despite this, most countries published fewer than 20 articles, indicating that many regions are still underrepresented in this field. In CiteSpace, larger circles represent countries with more publications, and lines between circles indicate collaboration (**Figure 3**). Countries with purple edges have high centrality, highlighting their importance in the network. Interestingly, while China, Japan, and South Korea contributed significantly in terms of publication volume, their centrality was relatively low. The Netherlands, despite fewer articles, showed high centrality due to extensive collaborations, reflecting different academic environments across Europe, America, and Asia.

**Table 1:** Top 10 countries/regions by publication count and centrality in PET/CT research in lymphoma.

Rank	Country	Count	Centrality	ACI	H-index	Institution	Count	Centrality	ACI	H-index
1	USA	684	0.40	36.76	67	UNICANCER(France)	126	0.08	31.90	37
2	CHINA	577	0.01	8.46	31	ASSISTANCE PUBLIQUE HOPITAUX PARIS APHP(France)	107	0.12	48.43	36
3	ITALY	274	0.20	31.83	45	UNIVERSITY OF LONDON(England)	96	0.05	87.42	36
4	FRANCE	264	0.03	31.70	49	INSTITUT NATIONAL DE LA SANTE ET DE LA RECHERCHE MEDICALE INSERM(France)	92	0.02	29.14	29
5	GERMANY	227	0.18	33.84	42	MEMORIAL SLOAN KETTERING CANCER CENTER(USA)	83	0.07	38.87	30
6	JAPAN	184	0.00	18.17	27	HARVARD UNIVERSITY(USA)	74	0.12	41.69	29
7	ENGLAND	165	0.09	58.39	42	UNIVERSITY OF TEXAS SYSTEM(USA)	73	0.03	34.42	27
8	SOUTH KOREA	140	0.00	25.10	30	UTMD ANDERSON CANCER CENTER(USA)	69	0.01	35.29	26
9	NETHERLANDS	115	0.15	37.05	31	SACKLER FACULTY OF MEDICINE(Israel)	57	0.07	28.74	22
10	SWITZERLAND	113	0.07	66.36	30	STANFORD UNIVERSITY(USA)	57	0.02	34.05	23

**Figure 4:**
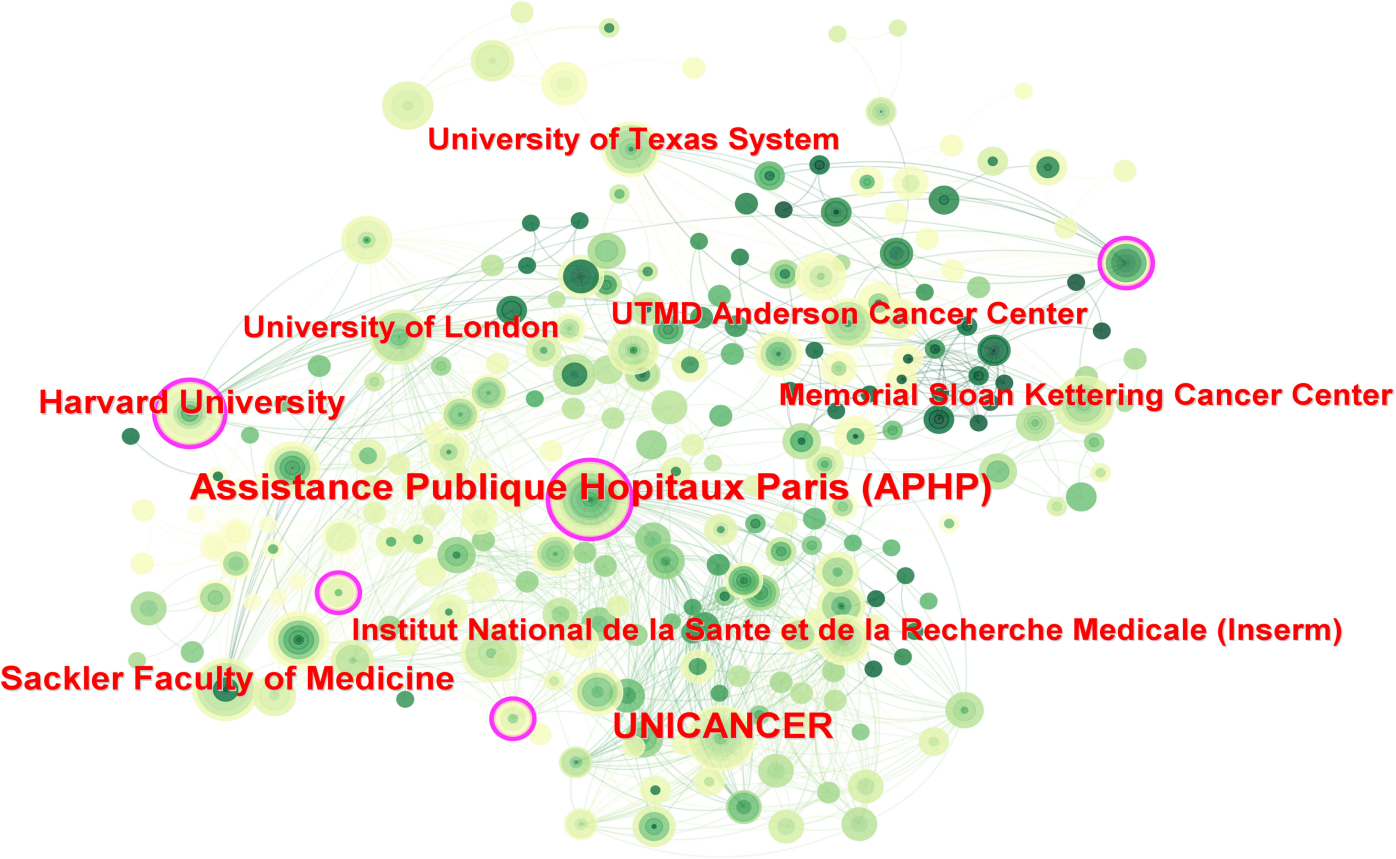
Inter-institutional cooperation network map. The size of the nodes indicates the number of publications, and the lines indicate collaboration frequency.

Over the past 20 years, more than 2,800 institutions have researched PET/CT in lymphoma. UNICANCER ranked first with 126 publications, followed by Assistance Publique Hôpitaux de Paris (APHP) with 107 articles, University of London with 96 articles, and INSERM with 92 publications. Among the top ten institutions, three were French, including the top two, and five were American (**Table 1**). Regarding institutional collaboration, higher centrality institutions (indicated by purple-edged circles) such as APHP, Memorial Sloan Kettering Cancer Center, and Harvard University were predominantly located in France and the USA (**Figure 4**).

Further analysis of publication types revealed distinct patterns in research output. For original research, the United States led with 611 publications, followed by China with 603 publications (**Supplementary Table 3**). A similar pattern was observed in review articles, with the United States contributing 152 reviews and China producing 87 reviews (**Supplementary Table 4**).

At the institutional level, Memorial Sloan Kettering Cancer Center demonstrated leadership in both original research (69 publications) and review articles (17 publications) (**Supplementary Table 4**). Chinese institutions, particularly Shanghai Jiao Tong University, showed strong performance in original research (57 publications) but had relatively lower representation in review publications (**Supplementary Table 4**).

### Authors and co-cited authors

**Figure 5:**
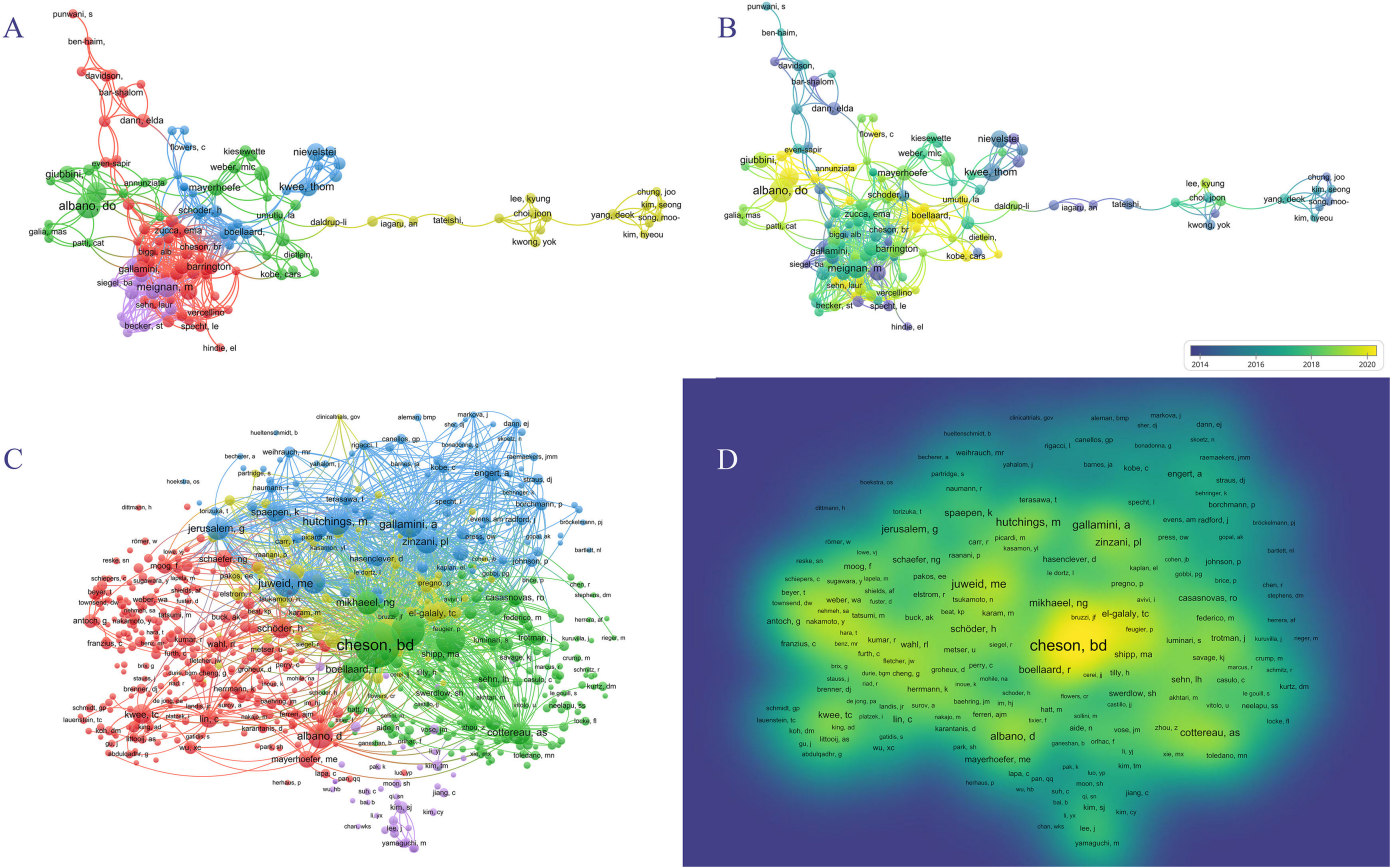
Author analyses in PET/CT research in lymphoma. **(A, B)** VOSviewer overlay visualization of authors. **(C)** The cluster graph showing the network of co-cited authors. **(D)** The density map of co-cited authors.

In the past 20 years, 15,149 authors have contributed to research on PET/CT in lymphoma. The top ten most prolific authors are listed in **Table 2**. Sally F. Barrington from King’s College London and Michel Meignan from Assistance Publique Hôpitaux de Paris led with 34 publications each, followed by Thomas C. Kwee from University of Groningen with 33 articles. Unlike institutional distribution, the top ten prolific authors came from seven different countries. Co-authorship networks divided into five groups, with the blue, green, and purple groups active post-2018, and the red and yellow groups prominent pre-2018 (**Figures 5A, B**). The most co-cited authors included Bruce Cheson from the Lymphoma Research Foundation with 1,587 citations, Sally F. Barrington with 860 citations, and Michel Meignan with 674 citations, demonstrating their significant influence in the field (**Table 2**). The citation relationships among co-cited authors are shown in the cluster graph, and the density map displays their influence in the field (**Figures 5C, D**).

**Table 2:** Top 10 authors by publication count and co-citations in PET/CT research in lymphoma.

Rank	Author	Institution	Publications	ACI	H-index	Co-cited author	Institution	Co-citations	ACI	H-index
1	Barrington, Sally F.	King's College London(England)	34	186.44	21	Cheson, Bruce	Lymphoma Res Fdn(USA)	1587	375.46	11
2	Meignan, Michel	Assistance Publique Hopitaux Paris (APHP)(French)	34	91.91	22	Barrington, Sally F.	King's College London(England)	860	186.44	21
3	Kwee, Thomas C.	University of Groningen(Netherlands)	33	34.36	15	Meignan, Michel	Assistance Publique Hopitaux Paris (APHP)(French)	674	91.91	22
4	Albano, Domenico	ASST Spedali Civili di Brescia(Italy)	31	17.9	14	Gallamini, Andrea	Centre Antoine Lacassagne(French)	564	41.52	16
5	Treglia, Giorgio	Ente Ospedaliero Cantonale, EOC(switzerland)	30	20.3	15	Hutchings, Martin	Rigshospitalet(Denmark)	547	91.55	19
6	Hutchings, Martin	Rigshospitalet(Denmark)	29	91.55	19	Adams, Hugo J. A.	University of Groningen(USA)	510	31.53	12
7	Gallamini, Andrea	Centre Antoine Lacassagne(French)	27	41.52	16	Albano, Domenico	ASST Spedali Civili di Brescia(Italy)	481	17.90	14
8	Schoder, Heiko	Memorial Sloan Kettering Cancer Center(USA)	25	31.44	16	Boellaard, Ronald	Locat Vrije Univ Amsterdam(Netherlands)	437	20.47	12
9	Giubbini, Raffaele	University of Brescia(Italy)	24	21.04	13	Kostakoglu, Lale	University of Virginia(USA)	428	66.26	14
10	Mayerhoefer, Marius	Cornell University(USA)	24	19.79	14	Mikhaeel, Nabegh George	King's College London(England)	425	105.38	11

### Journals and journal co-citation

**Figure 6:**
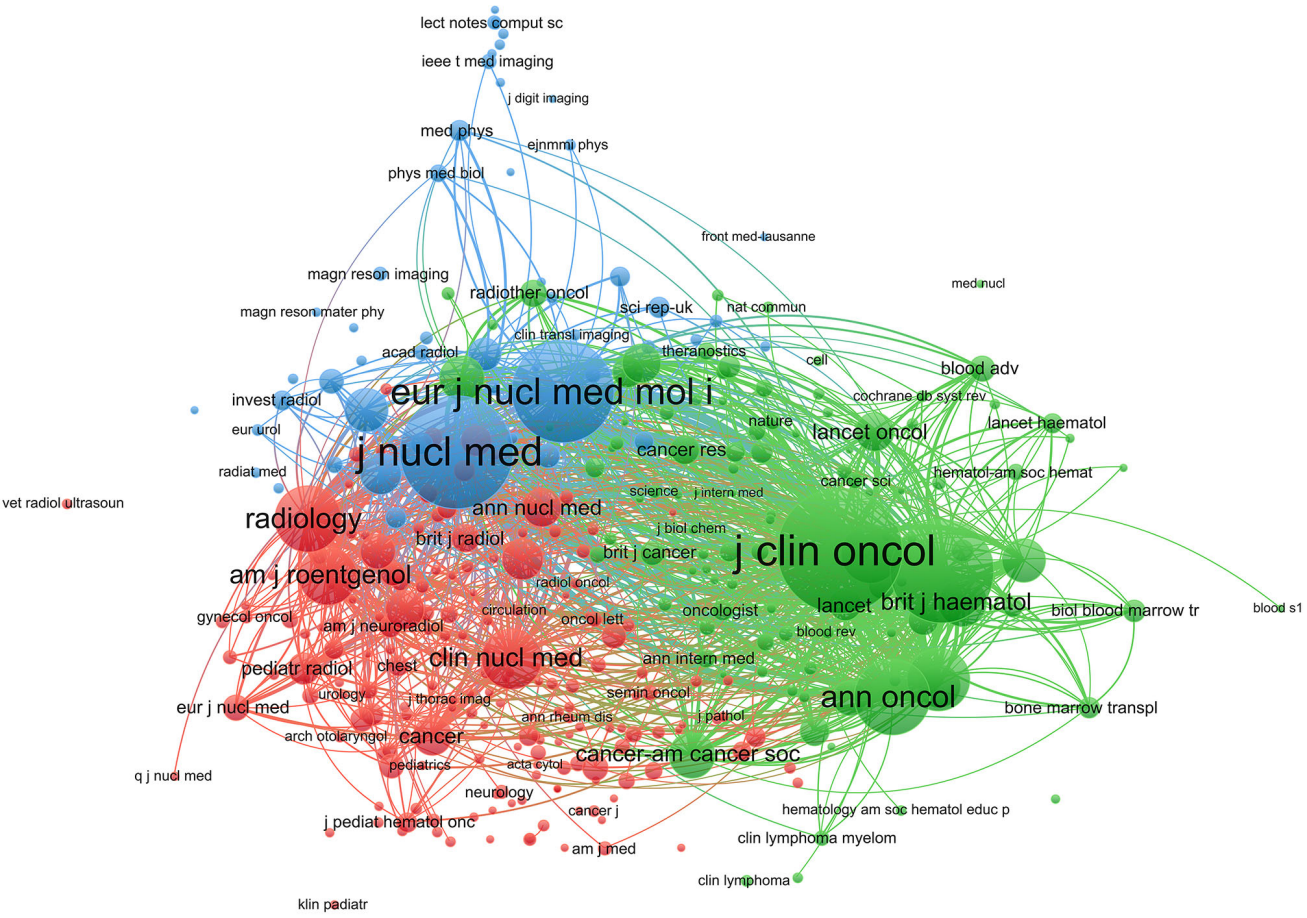
Co-citation network of journals in PET/CT research in lymphoma. Node size represents the number of citations, and lines represent co-citation relationships.

A total of 583 journals published research on PET/CT in lymphoma. The top ten journals are listed in **Table 3**, with the European Journal of Nuclear Medicine and Molecular Imaging leading with 152 articles (5.12%), followed by the Journal of Nuclear Medicine with 104 articles (3.50%) and Leukemia Lymphoma with 84 articles (2.83%). The top ten journals included four from the USA and two from the UK. Notably, the Annals of Nuclear Medicine from Japan contributed 58 articles. Among the top ten, three journals had an impact factor over 5, highlighting the high quality and significant impact of PET/CT research in lymphoma. Using VOSviewer, a co-citation analysis was conducted. Node size represents the number of citations, and lines between nodes represent co-citation relationships (**Figure 6**). The journals can be divided into three clusters: green for oncology journals, blue for nuclear medicine journals, and red for cancer and radiology journals. The Journal of Clinical Oncology (JCO) and the Journal of Nuclear Medicine (JNM) are the most authoritative journals in this field, with 8,759 and 7,155 co-citations, respectively. Among the top ten co-cited journals, eight have an impact factor above 9, and nine are in the Q1 category, including top-tier medical journals like the New England Journal of Medicine (**Table 4**). This bibliometric analysis identified 591 articles published in nuclear medicine journals, accounting for 19.96% of the total 2,962 studies analyzed. The European Journal of Nuclear Medicine and Molecular Imaging (173 articles) and the Journal of Nuclear Medicine (122 articles) emerged as the dominant contributors, reflecting their pivotal role in advancing PET/CT research for lymphoma. These journals also demonstrated high citation impact (e.g., Journal of Nuclear Medicine: 9,366 citations), underscoring their influence in both academic and clinical spheres.

**Table 3:** Top 10 journals by publication count and impact factor in PET/CT research in lymphoma.

Rank	Journal	Publications	Country	Impact factor	JCR	ACI	H-index
1	EUROPEAN JOURNAL OF NUCLEAR MEDICINE AND MOLECULAR IMAGING	152	GERMANY	9.1	Q1	40.51	47
2	JOURNAL OF NUCLEAR MEDICINE	104	UNITED STATES	9.3	Q1	78.26	42
3	LEUKEMIA LYMPHOMA	84	ENGLAND	2.6	Q3	14.81	21
4	NUCLEAR MEDICINE COMMUNICATIONS	81	UNITED STATES	1.5	Q4	15.42	19
5	CLINICAL NUCLEAR MEDICINE	70	UNITED STATES	10.6	Q1	20.40	22
6	MEDICINE	64	UNITED STATES	1.6	Q3	6.28	10
7	ANNALS OF NUCLEAR MEDICINE	58	JAPAN	2.6	Q3	17.78	21
8	ANNALS OF HEMATOLOGY	53	GERMANY	3.5	Q2	17.72	17
9	EUROPEAN JOURNAL OF RADIOLOGY	45	IRELAND	3.3	Q2	31.24	22
10	FRONTIERS IN ONCOLOGY	44	Switzerland	4.7	Q2	4.45	8

**Table 4:** Top 10 co-cited journals in PET/CT research in lymphoma.

Rank	Journal	Co-citations	Country	Impact Factor	JCR	ACI	H-index
1	JOURNAL OF CLINICAL ONCOLOGY	8759	UNITED STATES	45.3	Q1	339.41	15
2	JOURNAL OF NUCLEAR MEDICINE	7155	UNITED STATES	9.3	Q1	78.26	42
3	BLOOD	5927	UNITED STATES	20.3	Q1	83.26	18
4	EUROPEAN JOURNAL OF NUCLEAR MEDICINE AND MOLECULAR IMAGING	5759	GERMANY	9.1	Q1	40.51	47
5	ANNALS OF ONCOLOGY	3243	ENGLAND	50.5	Q1	63.42	17
6	RADIOLOGY	2449	UNITED STATES	19.7	Q1	79.07	20
7	LEUKEMIA LYMPHOMA	2192	ENGLAND	2.6	Q3	14.81	21
8	CLINICAL NUCLEAR MEDICINE	2026	UNITED STATES	10.6	Q1	20.40	22
9	NEW ENGLAND JOURNAL OF MEDICINE	1989	UNITED STATES	158.5	Q1	222.25	3
10	AMERICAN JOURNAL OF ROENTGENOLOGY	1860	UNITED STATES	5.0	Q1	41.76	22

### Co-cited references and clusters

**Figure 7:**
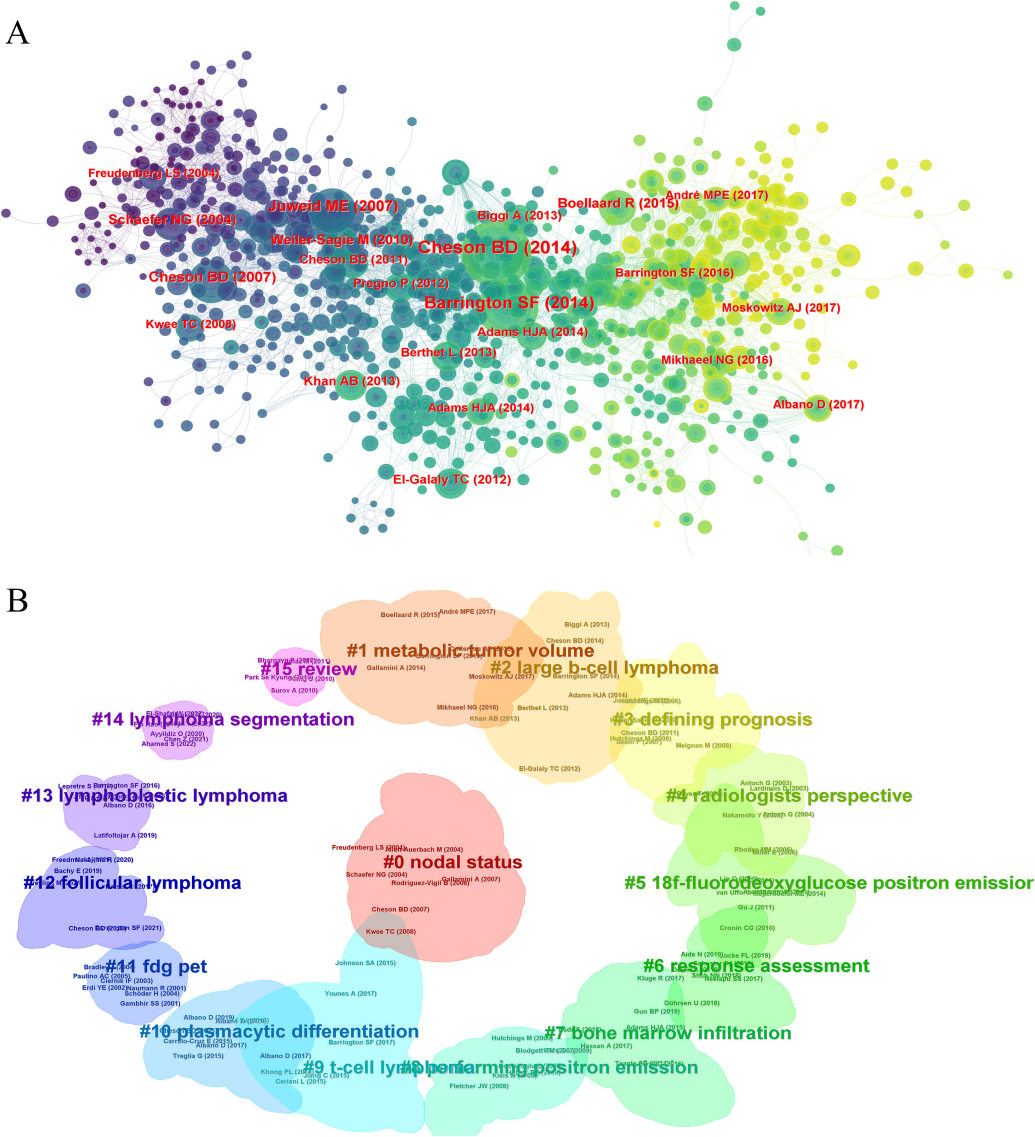
The journal and disciplinary analysis in PET/CT research in lymphoma. **(A)** The co-cited networks of the references. **(B)** The clustering analysis of the references.

Analyzing 928 co-cited references, the top ten are listed in **Table 5**. The most highly cited article is the “Recommendations for Initial Evaluation, Staging, and Response Assessment of Hodgkin and Non-Hodgkin Lymphoma: The Lugano Classification,” published in the Journal of Clinical Oncology in 2014, with 3,167 citations. This clinical guideline emphasizes the critical role of PET-CT in the diagnosis and management of lymphomas. PET-CT is considered an essential tool for initial staging and treatment response assessment because it provides detailed information about the metabolic activity of tumors, allowing for precise localization and evaluation of lymphomas. The guideline recommends the use of PET-CT both before and after treatment to determine the extent of disease spread and treatment efficacy. This imaging modality enhances the accuracy of staging and consistency of response assessment, making it an integral part of modern lymphoma management. The co-citation network in CiteSpace showed influential references (**Figure 7A**), and clustering analysis divided them into 16 related clusters (**Figure 7B**). These studies emphasized the importance of PET/CT in lymphoma diagnosis, staging, and treatment evaluation, particularly the use of 18F-FDG PET/CT. They explored personalized treatment adjustments based on PET/CT imaging to improve treatment outcomes and reduce unnecessary side effects, demonstrating the advancements in precision medicine. These studies provide a solid foundation for future research in precision medicine and personalized treatment.

**Table 5:** Top 10 co-cited references in PET/CT research in lymphoma.

Rank	Title	Citations	Year	Journal	Type	Impact factor
1	Recommendations for Initial Evaluation, Staging, and Response Assessment of Hodgkin and Non-Hodgkin Lymphoma: The Lugano Classification	3167	2014	JOURNAL OF CLINICAL ONCOLOGY	Article	45.3
2	From RECIST to PERCIST: Evolving Considerations for PET Response Criteria in Solid Tumors	2718	2009	JOURNAL OF NUCLEAR MEDICINE	Article	9.3
3	Role of Imaging in the Staging and Response Assessment of Lymphoma: Consensus of the International Conference on Malignant Lymphomas Imaging Working Group	1047	2014	JOURNAL OF CLINICAL ONCOLOGY	Article	45.3
4	Adapted Treatment Guided by Interim PET-CT Scan in Advanced Hodgkin's Lymphoma	537	2016	NEW ENGLAND JOURNAL OF MEDICINE	Article	158.5
5	Integrated PET/CT-3: Current applications and future directions	424	2006	RADIOLOGY	Article	19.7
6	PET/CT: Form and function	290	2007	RADIOLOGY	Review	19.7
7	Use of PET and PET/CT for Radiation Therapy Planning: IAEA expert report 2006-2007	286	2009	RADIOTHERAPY AND ONCOLOGY	Review	5.7
8	18F-FDG PET and PET/CT in fever of unknown origin	279	2007	JOURNAL OF NUCLEAR MEDICINE	Article	9.3
9	18F-FDG Avidity in Lymphoma Readdressed: A Study of 766 Patients	273	2010	JOURNAL OF NUCLEAR MEDICINE	Article	9.3
10	Non-Hodgkin lymphoma and Hodgkin disease: Coregistered EDG PET and CT at staging and restaging - Do we need contrast-enhanced CT?	252	2004	RADIOLOGY	Article	19.7

### Keywords

**Figure 8:**
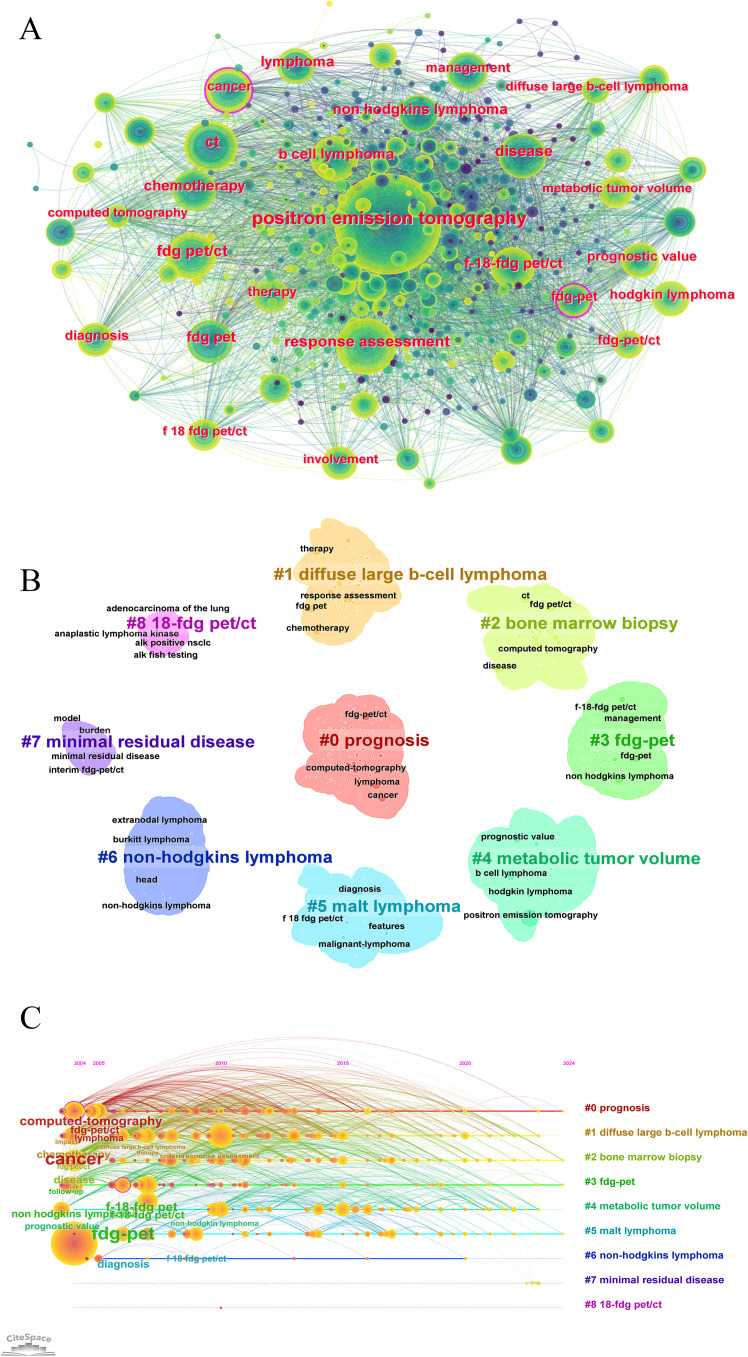
Keyword co-occurrence and trends analysis in PET/CT research in lymphoma. **(A)** Co-occurrence network analysis of keywords. **(B)** The clustering analysis of the keywords. **(C)** Timeline distribution of cluster analysis of the keywords.

Keyword analysis reflects current research themes, hotspots, and future directions. High-frequency keywords included b-cell lymphoma, chemotherapy, FDG-PET/CT, lymphoma, positron-emission-tomography, prognostic value, and response assessment, highlighting the core directions in PET/CT research in lymphoma (**Figure 8A**). These keywords underscore the application of PET/CT in diagnosis, treatment monitoring, and prognosis assessment, showing its critical role in lymphoma management. Keyword clustering divided the research into three groups, representing different research directions. The first group included Prognosis (#0), FDG-PET (#3), Metabolic Tumor Volume (#4), and 18-FDG PET/CT (#8), forming prognosis and diagnostic techniques. The second group covered Diffuse Large B-Cell Lymphoma (#1), MALT Lymphoma (#5), and Non-Hodgkin’s Lymphoma (#6), focusing on major lymphoma types. The third group included sBone Marrow Biopsy (#7) and Minimal Residual Disease (#6), indicating the advanced diagnostic and treatment monitoring. The average year of keyword occurrence was also visualized (**Figures 8B, C**).

### Non-FDG tracers analysis

While 18F-FDG remains the most investigated tracer, its limited sensitivity in indolent lymphomas (e.g., marginal zone lymphoma) and inflammatory false positives drive demand for targeted alternatives. Our literature search identified 84 studies investigating non-FDG tracers in lymphoma imaging. Non-FDG tracers ranked by frequency: CXCR4-targeted imaging (68Ga-Pentixafor, 35 studies) > FLT (29) > FAPI (9) > 18F-Fludarabine (12). Citation analysis of non-FDG tracer studies revealed 10 highly influential papers (**Supplementary Table 5**).

## Discussion

This study represents a comprehensive bibliometric analysis of PET/CT research in lymphoma, offering novel insights into the evolving trends, key contributors, and emerging research themes within this domain. The number of publications has steadily increased over the past two decades, peaking with a notable surge in 2021. This trend underscores the growing importance of PET/CT in lymphoma diagnosis and management. The citation counts also reflect a corresponding rise, indicating the increasing influence and recognition of this research within the scientific community. The projected growth in publications suggests that PET/CT will continue to be a focal point of lymphoma research, driven by ongoing advancements in imaging technology and its applications in personalized medicine.

The notable surge in publications during 2021 merits examination within the COVID-19 pandemic context. Several factors contributed to this increase: the pandemic accelerated telemedicine adoption, highlighting PET/CT’s value in providing comprehensive diagnostic information while minimizing patient contact (18); the need to evaluate COVID-19 complications in lymphoma patients prompted research into modified treatment protocols and response assessment strategies (19); and the maturation of pre-pandemic research projects, coupled with expedited peer review processes, likely contributed to increased publication output (20).

### Country, regional, institutional contributions, and author contributions

Our analysis identified the United States and China as the leading countries in PET/CT research, together accounting for a substantial portion of global publications. European countries such as Italy, France, and Germany also made significant contributions, although there remains a noticeable underrepresentation from other regions, highlighting a potential area for increased international collaboration. Institutions like UNICANCER and Assistance Publique Hôpitaux de Paris have been pivotal, producing a high volume of influential research. Notably, prolific authors such as Sally F. Barrington and Michel Meignan have played crucial roles in advancing the field, often working within these leading institutions (21, 22). The formation of research groups and networks, indicated by co-authorship patterns, underscores the collaborative nature of this research area and the geographic distribution of key contributors (4).

### Journals and journal co-citation analysis, and co-cited references

The European Journal of Nuclear Medicine and Molecular Imaging and the Journal of Nuclear Medicine emerged as the top journals publishing PET/CT research, reflecting their central role in disseminating high-impact studies. The co-citation analysis revealed distinct clusters of journals, with significant contributions from oncology, nuclear medicine, and radiology journals. This clustering indicates interdisciplinary collaboration and the integration of PET/CT research across various medical fields. The most co-cited references, such as guidelines on lymphoma staging and response assessment, highlight the critical contributions of PET/CT to precision medicine (3). Key guidelines like the Lugano Classification have been pivotal in standardizing the use of PET/CT for staging and response assessment in lymphoma, ensuring consistent and reliable interpretation of imaging results across different clinical settings (4, 23). These guidelines recommend PET/CT as the preferred imaging modality for initial staging and treatment response evaluation, reflecting its superior sensitivity and specificity in detecting both nodal and extranodal disease. The ability of PET/CT to provide detailed metabolic information allows for more accurate disease characterization, which is essential for tailoring treatment strategies to individual patient profiles (24).

### Co-cited references clusters and keyword analysis

The clustering of co-cited references and identification of high-frequency keywords reveal the core research themes in PET/CT applications for lymphoma. The prominence of keywords related to nuclear medicine applications and diagnostic techniques, such as “staging,” “response assessment,” “prognosis,” and “survival,” underscores the crucial role of PET/CT imaging in accurate disease characterization and monitoring. PET/CT’s ability to detect metabolic activity provides a more precise assessment of tumor burden compared to conventional imaging modalities like CT and MRI, which primarily rely on anatomical changes (25).

Staging and response assessment are critical components of lymphoma management. PET/CT is highly effective in differentiating between active disease and residual masses post-treatment, a common challenge in lymphoma care (26). This capability significantly influences prognosis and subsequent treatment decisions. Studies have shown that PET/CT-based response criteria, such as the Deauville five-point scale, are superior predictors of progression-free survival and overall survival compared to traditional methods (27). Additionally, parameters such as SUVmax, metabolic tumor volume (MTV), and total lesion glycolysis (TLG) are significant predictors of progression-free survival (PFS) and overall survival (OS) in patients with DLBCL (28, 29). Radiomic features extracted from PET/CT images, including texture and shape descriptors, have been used to build predictive models to predict treatment response and outcomes in lymphoma patients. These models demonstrate significant predictive capability and demonstrated high hazard ratios for predicting outcomes (30, 31).

Our bibliometric analysis reveals that 18F-FDG remains the dominant radiotracer in lymphoma PET/CT research, comprising over 97% of all studies identified. This predominance stems from FDG’s established role in detecting metabolically active disease in aggressive lymphoma subtypes and its extensive validation through clinical trials, which has led to its incorporation into standardized staging and response assessment frameworks such as the Lugano Classification and Deauville criteria (3, 27). Nevertheless, our analysis also identified emerging interest in novel non-FDG tracers, particularly CXCR4-targeted imaging agents. Among the 84 alternative radiotracer studies, CXCR4-targeted imaging (primarily 68Ga-Pentixafor) was most frequently investigated (35 publications), aligning with recent evidence demonstrating its utility in lymphoma subtypes with high CXCR4 expression, including marginal zone lymphoma and mantle cell lymphoma (32). 68Ga-Pentixafor PET/CT has demonstrated superior sensitivity in certain lymphoma subtypes, notably in MALT lymphoma and extranodal manifestations where FDG performance may be suboptimal (33).

Beyond FDG-PET/CT, these emerging non-FDG tracers demonstrate complementary potential in lymphoma imaging through targeting different biological processes. 18F-FLT, which targets cellular proliferation through thymidine kinase 1 activity, has shown particular value in therapy response assessment and early outcome prediction, especially in scenarios where FDG interpretation is challenged by inflammatory changes (34). The lymphoma-specific tracer 18F-Fludarabine, targeting the enzyme adenosine deaminase, has exhibited improved specificity compared to FDG in distinguishing lymphoma from inflammatory conditions, potentially reducing false-positive findings (35). More recently, preliminary studies with fibroblast activation protein inhibitor (FAPI) PET suggest potential value in detecting tumor-associated fibroblast activation within the lymphoma microenvironment (36). Despite these promising developments, our bibliometric analysis indicates that these novel tracers require further validation in larger cohorts and standardization of imaging protocols before widespread clinical implementation, reflecting the field’s progression toward more molecularly-targeted imaging approaches tailored to specific lymphoma subtypes and clinical contexts.

The clustering of keywords around specific lymphoma types, such as DLBCL, mucosa-associated lymphoid tissue (MALT) lymphoma, and non-Hodgkin’s lymphoma, reflects the diverse applications of PET/CT imaging across various lymphoma subtypes. Studies have demonstrated that interim PET/CT scans can predict treatment outcomes and guide therapeutic decisions in Hodgkin’s lymphoma and diffuse large B-cell lymphoma (DLBCL) (37–40). For MALT lymphoma and non-Hodgkin’s lymphoma, PET/CT helps in initial staging, treatment monitoring, and detection of residual disease, thereby improving overall management and prognosis (41, 42). The integration of PET/CT with advanced imaging techniques and biomarkers has significantly enhanced its role in lymphoma management. When combined with bone marrow biopsy, PET/CT provides comprehensive staging and diagnostic information (43, 44). Furthermore, PET/CT is crucial in detecting minimal residual disease (MRD), which is essential for assessing remission status and treatment response (45). The application of novel PET tracers and the integration of PET/CT with PET/MRI offer superior lesion characterization and multiparametric evaluation, enabling earlier detection and more accurate staging (46–48). As such, ongoing research and technological advancements in PET/CT imaging continue to shape the future of lymphoma management (49, 50).

The identification of these distinct clusters highlights the need for tailored imaging approaches and interpretation criteria to address the unique characteristics and therapeutic challenges associated with different lymphoma types. The emergence of keywords such as “immunotherapy,” “targeted therapy,” and “precision medicine” underscores the evolving landscape of lymphoma treatment and the integration of advanced therapeutic modalities. PET/CT imaging plays a pivotal role in guiding these novel treatment approaches by facilitating patient selection, monitoring treatment response, and identifying potential resistance mechanisms (51).

Immunotherapies and targeted therapies have revolutionized the treatment of lymphoma, offering more effective and less toxic options compared to traditional chemotherapy (52). PET/CT plays a pivotal role in these therapies by allowing precise assessment of tumor metabolism and early identification of metabolic changes during the treatment course. For instance, PET/CT is instrumental in evaluating the efficacy of therapies targeting specific molecular pathways, such as anti-CD20 monoclonal antibodies or checkpoint inhibitors (51). In patients undergoing sequential immunochemotherapy programs, such as rituximab plus R-CHOP, PET/CT can detect early metabolic responses that correlate with long-term clinical outcomes (53). What’s more, in clinical practice, PET/CT helps oncologists determine the suitability of patients for specific targeted treatments and monitor their response over time (54). Additionally, PET/CT can identify the development of resistance mechanisms, such as changes in tumor glucose metabolism, guiding subsequent therapeutic strategies (55).

CAR T-cell therapy represents a significant advancement in the treatment of lymphoma, particularly for patients with relapsed or refractory disease (56). PET/CT plays a crucial role in this therapeutic approach by providing detailed metabolic information that assists in patient selection and monitoring treatment response. The ability of PET/CT to detect metabolic changes early in the treatment course is particularly beneficial in assessing the effectiveness of CAR T-cell therapy and identifying potential resistance mechanisms (57). This integration of PET/CT with CAR T-cell therapy exemplifies the shift towards more personalized and precise treatment strategies in lymphoma care (58).

### Future research directions

The integration of artificial intelligence (AI) and machine learning techniques with PET/CT imaging holds immense potential for enhancing diagnostic accuracy, treatment response evaluation, and prognostic prediction in lymphoma (59). AI algorithms can assist in lesion detection, segmentation, and quantification, as well as in the extraction of complex imaging features that may be predictive of treatment outcomes (60, 61). Beyond image analysis, AI advances have revolutionized PET/CT procedures through low-dose protocols (62), ultra-fast scanning techniques (63), and enhanced image registration accuracy (64), simultaneously improving diagnostic quality while reducing radiation exposure and examination time. With ongoing standardization and validation of AI methodologies, AI is poised to become integral to both PET/CT scanning procedures and clinical interpretation in lymphoma care, enabling more personalized treatment decisions.

Furthermore, the development of novel PET tracers and imaging modalities, such as new radiopharmaceuticals with the identification of pathways or specific receptors in lymphomas, could provide more comprehensive and personalized assessments of lymphoma, enabling earlier detection and more accurate staging (65, 66). Prospective studies focusing on the role of PET/CT in guiding targeted therapies and immunotherapies for lymphoma are warranted, as these treatment approaches continue to evolve and gain wider acceptance. Longitudinal studies examining the long-term impact of PET/CT-guided management strategies on patient outcomes and quality of life could further inform clinical decision-making and contribute to the refinement of treatment protocols. Multicenter collaborative efforts and the standardization of PET/CT imaging protocols and response criteria are essential to facilitate data sharing, enable large-scale analyses, and ultimately improve the reproducibility and generalizability of findings. Harmonization of imaging practices and the establishment of consensus guidelines can enhance the comparability of results across institutions and pave the way for more robust clinical trials and evidence-based decision-making.

### Limitations of the study

This bibliometric analysis presents several limitations. Firstly, potential biases in the data source and search strategy might have influenced the results, as our reliance on the Web of Science Core Collection database may exclude relevant studies published elsewhere. Moreover, our analysis is constrained by the inherent limitations of bibliometric methodologies, which often cannot fully capture the complexity of interdisciplinary collaborations or the nuanced impact of individual studies. Furthermore, a significant limitation is our exclusive use of traditional citation metrics without incorporating altmetrics data. Although altmetrics provides valuable insights into immediate social and academic impact, several factors hindered its implementation: technical challenges with WoS data integration, inherent bias toward recent publications, and absence of field-specific benchmarking standards. Consequently, future analyses should aim to synthesize traditional metrics with altmetrics for a more comprehensive assessment of research impact. Additionally, the histopathologic heterogeneity across lymphoma subtypes (e.g., Hodgkin vs. DLBCL) may confound PET/CT interpretation and research comparability, thus necessitating future subtype-stratified analyses that account for the distinct biological and imaging characteristics of major lymphoma categories. Finally, the dynamic and rapidly advancing nature of PET/CT technology requires ongoing updates to capture emerging developments and trends. Therefore, subsequent research endeavors should address these limitations through more comprehensive data collection methodologies and diversified analytical approaches that better reflect the evolving landscape of PET/CT applications in lymphoma management.

## Conclusion

In conclusion, this study provides a comprehensive overview of the research trends in PET/CT for lymphoma, highlighting the significant contributions and growing importance of this field. The findings align with the initial research goals, emphasizing the role of PET/CT in advancing personalized medicine and improving clinical outcomes in lymphoma. The study underscores the need for continued research and collaboration, particularly in emerging areas like AI integration. The insights gained from this analysis can inform future research directions and clinical practices, ultimately enhancing the management and treatment of lymphoma patients.

## Data Availability

The original contributions presented in the study are included in the article/**Supplementary Material**. Further inquiries can be directed to the corresponding authors.
