# Supplementary material for: Mapping the research landscape of PET/CT in lymphoma: insights from a bibliometric analysis
Source: Front Oncol. 2025 Apr 8;15:1513296. doi: 10.3389/fonc.2025.1513296 (PMC12011559; doi:10.3389/fonc.2025.1513296)
Supplement: Supplementary file 1 [file DataSheet1.docx]

Supplementary Table 1. Top 10 highly cited multicenter studies

| **Rank** | **Title** | **Journals** | **First author** | **Year** | **citations** |
| --- | --- | --- | --- | --- | --- |
| 1 | Baseline Metabolic Tumor Volume Predicts Outcome in High-Tumor-Burden Follicular Lymphoma: A Pooled Analysis of Three Multicenter Studies | JOURNAL OF CLINICAL ONCOLOGY | Meignan, M | 2016 | 218 |
| 2 | Definitive radiotherapy for localized follicular lymphoma staged by 18F-FDG PET-CT: a collaborative study by ILROG | BLOOD | Brady, JL | 2019 | 86 |
| 3 | The number of extranodal sites assessed by PET/CT scan is a powerful predictor of CNS relapse for patients with diffuse large B-cell lymphoma: An international multicenter study of 1532 patients treated with chemoimmunotherapy | EUROPEAN JOURNAL OF CANCER | El-Galaly | 2017 | 69 |
| 4 | The role of bone marrow biopsy and FDG-PET/CT in identifying bone marrow infiltration in the initial diagnosis of high grade non-Hodgkin B-cell lymphoma and Hodgkin lymphoma. accuracy in a multicenter series of 372 patients | AMERICAN JOURNAL OF HEMATOLOGY | Chen-Liang, TH | 2015 | 44 |
| 5 | Diffuse Large B-Cell Lymphoma: Prospective Multicenter Comparison of Early Interim FLT PET/CT versus FDG PET/CT with IHP, EORTC, Deauville, and PERCIST Criteria for Early Therapeutic Monitoring | RADIOLOGY | Minamimoto, R | 2016 | 40 |
| 6 | Whole-Body MRI-DWI for Assessment of Residual Disease after Completion of Therapy in Lymphoma: A Prospective Multicenter Study | JOURNAL OF MAGNETIC RESONANCE IMAGING | Littooij, AS | 2015 | 33 |
| 7 | Diagnostic and prognostic value of 18F-FDG PET/CT in comparison with morphological imaging in primary adrenal gland malignancies - a multicenter experience | HELLENIC JOURNAL OF NUCLEAR MEDICINE | Cistaro, A | 2015 | 26 |
| 8 | Multicenter Comparison of Contrast-Enhanced FDG PET/CT and 64-Slice Multi-Detector-Row CT for Initial Staging and Response Evaluation at the End of Treatment in Patients With Lymphoma | CLINICAL NUCLEAR MEDICINE | León, NG | 2017 | 24 |
| 9 | FDG PET/CT to detect bone marrow involvement in the initial staging of patients with aggressive non-Hodgkin lymphoma: results from the prospective, multicenter PETAL and OPTIMAL>60 trials | EUROPEAN JOURNAL OF NUCLEAR MEDICINE AND MOLECULAR IMAGING | Kaddu-Mulindwa, D | 2021 | 23 |
| 10 | [18F]-FDG PET/CT in the Staging and Management of Indolent Lymphoma: A Prospective Multicenter PET Registry Study | CANCER | Metser, U | 2017 | 23 |

Supplementary Table 2. Top 10 highly cited single-center studies

| **Rank** | **Title** | **Journals** | **First author** | **Year** | **citations** |
| --- | --- | --- | --- | --- | --- |
| 1 | 18F-FDG Avidity in Lymphoma Readdressed: A Study of 766 Patients | JOURNAL OF NUCLEAR MEDICINE | Weiler-Sagie, M | 2010 | 285 |
| 2 | PET-CT staging of DLBCL accurately identifies and provides new insight into the clinical significance of bone marrow involvement | BLOOD | Khan, AB | 2013 | 176 |
| 3 | 18F-FDG PET/CT Uptake Classification in Lymphoma and Lung Cancer by Using Deep Convolutional Neural Networks | RADIOLOGY | Sibille, L | 2020 | 140 |
| 4 | Ionising radiation-free whole-body MRI versus 18F-fluorodeoxyglucose PET/CT scans for children and young adults with cancer: a prospective, non-randomised, single-centre study | LANCET ONCOLOGY | Klenk, C | 2014 | 122 |
| 5 | The Role of 18F-FDG PET/CT for Initial Staging of Nasal Type Natural Killer/T-Cell Lymphoma: A Comparison with Conventional Staging Methods | JOURNAL OF NUCLEAR MEDICINE | Moon, SH | 2013 | 60 |
| 6 | Staging accuracy in mycosis fungoides and Sezary syndrome using integrated positron emission tomography and computed tomography | ARCHIVES OF DERMATOLOGY | Tsai, EY | 2006 | 60 |
| 7 | Midtreatment 18F-FDG PET/CT Scan for Early Response Assessment of SMILE Therapy in Natural Killer/T-Cell Lymphoma: A Prospective Study from a Single Center | JOURNAL OF NUCLEAR MEDICINE | Khong, PL | 2014 | 56 |
| 8 | Diagnosis of intravascular large B cell lymphoma: novel insights into clinicopathological features from 42 patients at a single institution over 20 years | BRITISH JOURNAL OF HAEMATOLOGY | Matsue, K | 2019 | 52 |
| 9 | Single-Institution Experience in the Treatment of Primary Mediastinal B Cell Lymphoma Treated With Immunochemotherapy in the Setting of Response Assessment by 18Fluorodeoxyglucose Positron Emission Tomography | INTERNATIONAL JOURNAL OF RADIATION ONCOLOGY BIOLOGY PHYSICS | Pinnix, CC | 2015 | 45 |
| 10 | Efficacy of routine surveillance with positron emission tomography/computed tomography in aggressive non-Hodgkin lymphoma in complete remission: status in a single center | LEUKEMIA & LYMPHOMA | El-Galaly, T | 2011 | 38 |

Supplementary Table 3. Distribution of original research among top 10 countries and institutions

| **Rank** | **Country** | **Documents** | **Citations** | **TLS** | **Rank** | **Organization** | **Documents** | **Citations** | **TLS** |
| --- | --- | --- | --- | --- | --- | --- | --- | --- | --- |
| 1 | USA | 611 | 24632 | 413 | 1 | Memorial Sloan Kettering Cancer Center | 69 | 2536 | 170 |
| 2 | China | 603 | 5514 | 108 | 2 | Shanghai Jiao Tong University | 57 | 528 | 39 |
| 3 | Italy | 245 | 7825 | 263 | 3 | Tel Aviv University | 49 | 1640 | 86 |
| 4 | Japan | 219 | 3964 | 63 | 4 | The University of Texas MD Anderson Cancer Center | 48 | 1542 | 89 |
| 5 | Germany | 216 | 7104 | 256 | 5 | Stanford University | 46 | 1820 | 87 |
| 6 | France | 207 | 7865 | 222 | 6 | Sun Yat-sen University | 42 | 390 | 24 |
| 7 | South Korea | 169 | 3973 | 65 | 7 | Sichuan University | 42 | 395 | 16 |
| 8 | England | 132 | 9275 | 225 | 8 | Fudan University | 40 | 434 | 33 |
| 9 | Switzerland | 105 | 8206 | 228 | 9 | Medical University of Vienna | 38 | 672 | 69 |
| 10 | Netherlands | 100 | 3429 | 153 | 10 | Mayo Clinc | 38 | 867 | 63 |

Supplementary Table 4. Distribution of review among top 10 countries and institutions

| **Rank** | **Country** | **Documents** | **Citations** | **TLS** | **Rank** | **Organization** | **Documents** | **Citations** | **TLS** |
| --- | --- | --- | --- | --- | --- | --- | --- | --- | --- |
| 1 | USA | 152 | 4646 | 90 | 1 | Memorial Sloan Kettering Cancer Center | 17 | 797 | 8 |
| 2 | China | 87 | 919 | 4 | 2 | Zhejiang University | 14 | 110 | 0 |
| 3 | Italy | 77 | 2315 | 99 | 3 | Mayo Clinc | 12 | 131 | 9 |
| 4 | England | 44 | 1537 | 67 | 4 | University of Brescia | 11 | 105 | 21 |
| 5 | Germany | 43 | 2118 | 70 | 5 | The University of Texas MD Anderson Cancer Center | 11 | 350 | 9 |
| 6 | France | 42 | 1587 | 66 | 6 | Harvard Medical School | 10 | 136 | 10 |
| 7 | Netherlands | 33 | 1499 | 64 | 7 | Massachusetts General Hospital | 10 | 535 | 9 |
| 8 | Australia | 23 | 1223 | 49 | 8 | Harvard University | 9 | 494 | 5 |
| 9 | Belgium | 23 | 1028 | 48 | 9 | King's College London | 9 | 441 | 1 |
| 10 | Switzerland | 22 | 290 | 31 | 10 | Brigham and Women's Hospital | 8 | 112 | 16 |

Supplementary Table 5. Top 10 highly cited articles focusing on non-FDG tracers

| **Rank** | **Title** | **Journals** | **First author** | **Year** | **citations** |
| --- | --- | --- | --- | --- | --- |
| 1 | A systematic review on [18F]FLT-PET uptake as a measure of treatment response in cancer patients | EUROPEAN JOURNAL OF CANCER | Bollineni, VR | 2016 | 82 |
| 2 | Molecular PET and PET/CT imaging of tumour cell proliferation using F-18 fluoro-L-thymidine: a comprehensive evaluation | NUCLEAR MEDICINE COMMUNICATIONS | Barwick, T | 2009 | 78 |
| 3 | CXCR4-targeted theranostics in oncology | EUROPEAN JOURNAL OF NUCLEAR MEDICINE AND MOLECULAR IMAGING | Buck, AK | 2022 | 66 |
| 4 | Imaging of C-X-C Motif Chemokine Receptor 4 Expression in 690 Patients with Solid or Hematologic Neoplasms Using 68Ga-Pentixafor PET | JOURNAL OF NUCLEAR MEDICINE | Buck, AK | 2022 | 65 |
| 5 | The potential utility of [68 Ga]Ga-DOTA-FAPI-04 as a novel broad-spectrum oncological and non-oncological imaging agent-comparison with [18F]FDG | EUROPEAN JOURNAL OF NUCLEAR MEDICINE AND MOLECULAR IMAGING | Lan, LJ | 2022 | 65 |
| 6 | Predictive Value of Initial 18F-FLT Uptake in Patients with Aggressive Non-Hodgkin Lymphoma Receiving R-CHOP Treatment | JOURNAL OF NUCLEAR MEDICINE | Herrmann, K | 2011 | 57 |
| 7 | 68Ga-Pentixafor PET/CT for Imaging of Chemokine Receptor 4 Expression in Waldenstrom Macroglobulinemia/Lymphoplasmacytic Lymphoma: Comparison to 18F-FDG PET/CT | JOURNAL OF NUCLEAR MEDICINE | Luo, YP | 2019 | 44 |
| 8 | Detecting Fibroblast Activation Proteins in Lymphoma Using 68Ga-FAPI PET/CT | JOURNAL OF NUCLEAR MEDICINE | Jin, X | 2022 | 42 |
| 9 | Improved Primary Staging of Marginal-Zone Lymphoma by Addition of CXCR4-Directed PET/CT | JOURNAL OF NUCLEAR MEDICINE | Duell, J | 2021 | 42 |
| 10 | Diffuse Large B-Cell Lymphoma: Prospective Multicenter Comparison of Early Interim FLT PET/CT versus FDG PET/CT with IHP, EORTC, Deauville, and PERCIST Criteria for Early Therapeutic Monitoring | RADIOLOGY | Minamimoto, R | 2016 | 40 |
